# Safety and Efficacy of Octyl 2-Cyanoacrylate (Dermabond) in Breast Surgery: A Systematic Review

**DOI:** 10.3390/jcm15062462

**Published:** 2026-03-23

**Authors:** Maciej Rybicki, Marta Fijałkowska, Anna Kasielska-Trojan

**Affiliations:** Plastic, Reconstructive and Aesthetic Surgery Clinic, Institute of Surgery, Medical University of Lodz, 90-419 Lodz, Poland; fijalkowska.m@wp.pl

**Keywords:** breast surgery, complications, cosmetic outcome, cyanoacrylate tissue adhesive, dermabond, octyl 2-cyanoacrylate, wound closure

## Abstract

**Background/Objectives:** Effective wound closure in breast surgery achieves a compromise between safety and aesthetic outcomes. This systematic review evaluates the effect of 2-octyl cyanoacrylate (2-OCA) tissue adhesive (Dermabond) as a primary means of skin closure compared with traditional sutures. **Methods:** The study protocol was registered in PROSPERO (CRD42023420595). A systematic search of PubMed, Embase, and Web of Science databases identified ten studies (*n* = 1870) comparing 2-OCA with standard suturing techniques in breast reduction, reconstruction, and oncological procedures. The methodological quality was assessed using Cochrane RoB 2.0 and the Newcastle–Ottawa Scale. **Results:** Using the Synthesis Without Meta-analysis (SWiM) framework, the results indicate that 2-OCA offers a safety profile comparable to sutures in terms of the incidence of infection and hematoma. Although a higher incidence of wound dehiscence was observed with glue, 2-OCA showed better operative efficacy and greater patient satisfaction, which is attributed to its immediate water resistance and elimination of the need for suture removal. **Conclusions:** The analysis suggests that 2-OCA is a feasible structural alternative to skin sutures in appropriately selected patients, with the proper management of deep skin layer tension.

## 1. Introduction

Breast surgery is performed for a range of oncological, reconstructive, functional, and esthetic purposes, and is now an integral part of contemporary surgical practice. Despite its routine nature, it continues to pose a serious clinical challenge, particularly in terms of ensuring reliable wound healing and favorable cosmetic results. For many patients, especially those undergoing reduction or reconstruction procedures, minimizing postoperative complications remains a key factor in the overall success of treatment.

Postoperative complications also remain a frequent problem, especially after reconstructive and reduction procedures, which are associated with a higher risk of blood circulation disorders and tissue tension [1,2]. Such incidents can result in delayed recovery, the need for additional interventions or deterioration of the esthetic outcome, which may significantly affect the final result of the treatment. This is particularly important in oncology patients, for whom delayed ongoing treatment may significantly worsen the prognosis and chances of full recovery [3,4].

Hence, in patients undergoing breast reduction or reconstruction, a crucial element of treatment remains related to minimizing postoperative complications. The risk of healing disorders is strongly influenced by both patient-related factors (age, obesity, diabetes, and smoking) and surgical factors (extent of resection and quality of the skin flap) [2,5,6]. Their incidence can be reduced by correct early identification, the implementation of preventive strategies, and appropriate patient education. As proper healing is also strongly influenced by the method of wound closure and management, this represents an important consideration in modern surgery.

One approach to wound closure involves the use of cyanoacrylate-based substances; these were first developed in 1949 and were used as alternatives to sutures for the preparation of minor wounds a decade later [7,8,9]. One of the most well-established cyanoacrylate-derived tissue adhesives is 2-octyl cyanoacrylate (2-OCA), which is commonly used in surgery to close postoperative wounds [10,11]. 2-OCA can be used as a superficial dressing equivalent or an adjunct to superficial skin closure, rather than a substitute for deep dermal sutures. Due to the efficient anionic polymerization process that occurs upon contact with the moist skin surface, 2-OCA forms durable covalent bonds with the stratum corneum. This creates a waterproof and flexible protective layer that keeps the edges of the wound tightly together and effectively protects against external factors [10,12,13]. When compared to 5–0 sutures, the wound edge retention strength is comparable, and the application time is significantly shorter [10,12,14]; 2-OCA exhibits antibacterial properties and does not require removal, which increases patient comfort [12,15,16]. Also, the use of adhesive reduces the risk of accidental needle puncture among operative personnel [10,17,18,19].

In breast surgery, wound closure is most commonly performed using skin sutures, staplers, and adhesive tapes such as Steri-Strips. Each of these techniques has its advantages and limitations [19]. Sutures require removal, may leave a visible mark, and may be considered a risk factor for poor scar appearance (e.g., hypertrophic scarring/keloids) [20]. Staplers are quick and easy to use, but their removal can be painful, and the cosmetic effect is often considered less satisfactory. Steri-Strips and surgical tapes are non-invasive; however, they do not always allow the edges of the wound to be brought together properly, especially in high-tension skin areas [19,21].

One attractive alternative is Dermabond, a 2-OCA-based tissue adhesive which appears to offer a number of benefits. Most importantly, it has rapid application: the time needed to apply tissue adhesive is typically seven minutes shorter than sutures; the resulting coating is also waterproof, allowing patients to start bathing as early as 24 h after surgery [11,22]. The adhesive does not require removal, as the coating dissolves spontaneously and typically falls off within five to ten days. Cosmetic outcomes are often superior, with 95% of patients rating the esthetic effect as at least good, and postoperative pain tends to be reduced. While evidence from other surgical fields suggests a lower risk of infection with tissue adhesives, its specific impact in breast surgery requires focused evaluation [11,23,24,25].

The evaluation of the effectiveness of tissue adhesives in breast surgery must take into account the various surgical techniques used in procedures, as these are the main determinants of the biomechanical load on the incision lines.

As an example, the most popular breast reduction techniques include the “skin brassiere” and the “pillar-based” technique. The former uses the skin envelope as the primary structural support for the remaining mammary gland, which results in high tension on the skin closure. This can test the tensile strength of surface adhesives [10,16,26,27]. Conversely, the pillar-based technique focuses on the internal modeling of the breast tissue. Proper suturing of the glandular pillars is intended to support the breast, thereby transferring the load to the deep tissues. As a result, the skin closure is exposed to significantly less tension than with the skin brassiere technique [10,15,25,26]. Nevertheless, there is a lack of direct studies analyzing the impact of skin tension on the effectiveness of 2-OCA-based adhesives in breast reduction.

The purpose of this review is to provide an overview of the current knowledge regarding the application, effects and complications of Dermabond use in breast surgery.

## 2. Materials and Methods

This systematic review was conducted using a validated methodology, in accordance with the guidelines outlined in the Preferred Reporting Items for Systematic Reviews and Meta-Analyses (PRISMA) statement. The fully completed PRISMA checklist is included in the Supplementary Materials as Supplementary Table S1 [27]. The protocol for this systematic review was registered in the PROSPERO database (registration number: CRD42023420595).

### 2.1. Sources of Information and Search Strategy

A systematic literature search was conducted in line with PRISMA guidelines. Four primary electronic databases were interrogated: PubMed (MEDLINE), Embase (Ovid platform), Web of Science, and ClinicalTrials.gov. Additional searches were performed in the WHO International Clinical Trials Registry Platform (ICTRP). Further sources were identified by manually screening the reference lists from the included articles.

The strategies integrated both MeSH terms and free-text keywords relating to the intervention (octyl cyanoacrylate, 2-octyl cyanoacrylate, and Dermabond) and the surgical setting (mammaplasty, breast reconstruction, breast implantation, breast prosthesis, breast implant, mastopexy, breast conservation surgery, breast oncoplasty, oncoplastic surgery, and breast deformity). Boolean operators and truncation symbols were adapted to the requirements of each database.

PubMed (MEDLINE): The search combined MeSH headings and text words (e.g., “octyl cyanoacrylate” [tw] and “Dermabond” [tw]) with breast surgery-related terms (“Mammaplasty” [mh], “Breast Implantation” [mh], “oncoplastic surgery”, etc.). The initial search on 10 March 2023 returned 12 records, and the update on 3 September 2025 yielded 16 records.

Embase (Ovid): A structured search combining intervention-related and breast surgery-related domains produced 25 results, narrowed to 16 after excluding conference abstracts (17 March 2023).

Web of Science: Topic searches using synonymous terms yielded four results (20 March 2023), with eight results obtained in an updated search on the same date.

ClinicalTrials.gov: The query (octyl 2-cyanoacrylate OR Dermabond) AND breast identified two trials (NCT00558246 and NCT00890578), with additional studies located in subsequent updates (NCT05508945, NCT04321967, and NCT02779296).

WHO ICTRP: The search Dermabond AND breast identified relevant interventional studies, including ISRCTN30055885 (“the role of PREVENA vacuum dressings in patients undergoing breast surgery affecting both sides”).

The search was restricted to peer-reviewed, full-text articles in English published until 3 September 2025 (final update). Non-English manuscripts, abstracts, and gray literature were excluded.

All search results were exported into a reference management system; duplicates were removed, and the records underwent a two-stage screening (title/abstract, followed by full text). To minimize omissions, supplementary manual searches of reference lists and citation tracking were also performed. The details of the databases searched, dates, search terms, and eligibility criteria are summarized in Supplementary Table S2.

The inclusion criteria comprised original clinical trials (randomized controlled trials and observational studies) and systematic reviews published in peer-reviewed journals, written in English, and indexed in the above-mentioned databases. Manuscripts in languages other than English, conference abstracts, case reports, letters, editorials, and animal studies were all excluded.

**Figure 1:**
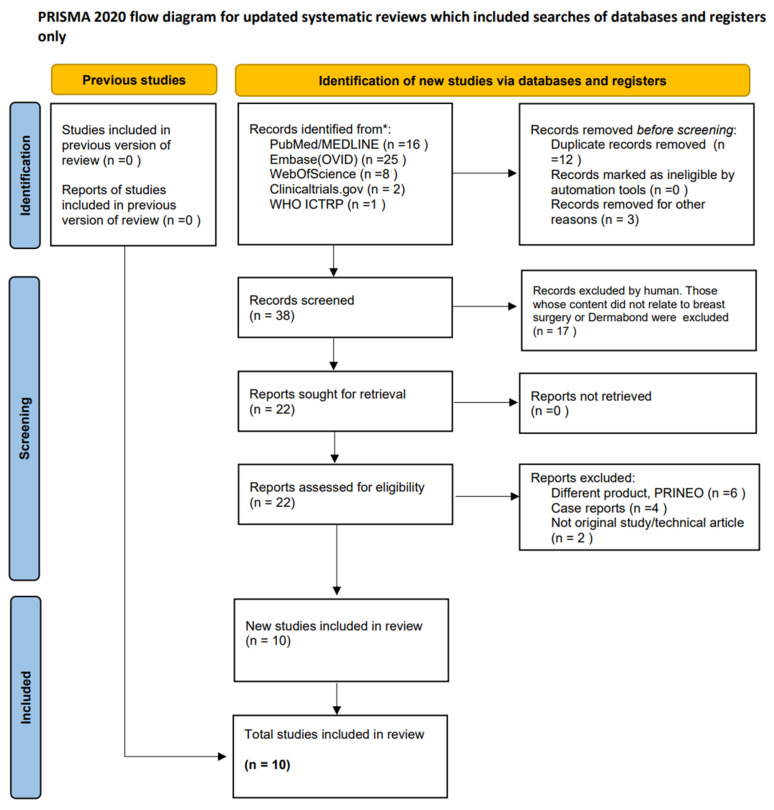
PRISMA 2020 diagram showing the selection of studies for the systematic review.Source: [27]. This work is licensed under a Creative Commons Attribution 4.0 International License (CC BY 4.0). To view a copy of this license, visit https://creativecommons.org/licenses/by/4.0/ (accessed on 12 January 2026)”. * A search of the databases was performed on 10 March 2025.

The selection process for the studies is shown in Figure 1 in the form of a PRISMA 2020 diagram. Finally, ten studies meeting the established eligibility criteria were included in the systematic review.

During the systematic search, a total of 52 records were identified in various databases: 15 in PubMed, 25 in Embase, two in ClinicalTrials.gov and one in WHO ICTRP. The Web of Science database did not contain any additional publications. After removing duplicates, 38 unique records remained, which were then preliminarily assessed based on the titles and abstracts.

At this stage, 16 publications were excluded, and 22 full-text articles were selected for further analysis based on the content of the paper. As a result, 12 papers were excluded from the study: six articles were concerned with a different product (PRINEO), four were case reports, and two were not original scientific studies (technical or review articles).

### 2.2. Research Selection, Data Gathering and Collection Process

All records identified during the database search were exported to a reference management system and then analyzed using Rayyan. A two-stage selection process was used. First, two reviewers independently assessed titles and abstracts against predefined eligibility criteria. Discrepancies were resolved through discussion, and in cases where a consensus could not be reached, a third reviewer was consulted. In the second stage, the same reviewers assessed the full texts of potentially relevant articles.

The following elements were extracted from the included manuscripts: bibliographic data, study design, patient demographics, surgical context, characteristics of the intervention and comparison, outcome measures, and methodological quality indicators. Any discrepancies during data extraction were resolved by consensus. The data were extracted independently and twice using a predefined Excel spreadsheet.

This structured approach ensured reproducibility during data selection and collection, and minimized the risk of bias.

### 2.3. Data Items

The primary outcome was the cosmetic appearance of the postoperative wound. Supplementary outcomes ranged from the incidence of surgical wound infection to the duration of use, postoperative pain, wound dehiscence, and patients’ satisfaction.

### 2.4. Risk of Systematic Bias in Particular Studies

The risk of bias in randomized controlled trials was assessed using the Cochrane Risk of Bias 2.0 (RoB 2) tool. This method was applied to four studies ([11,22,28,29] Observational studies were assessed using the Newcastle–Ottawa Scale (six papers) [30,31,32,33,34,35]. Two reviewers independently assessed the risk of bias, and discrepancies were resolved through discussion or arbitration by a third reviewer.

### 2.5. Effect Measures and Synthesis Methods

Due to the considerable heterogeneity of studies’ designs, interventions, and reporting of results, it was not possible to conduct an objective meta-analysis. Therefore, data synthesis was conducted in accordance with the Synthesis Without Meta-analysis (SWiM) reporting guidelines. We utilized a structured approach to tabulate the studies’ characteristics and outcomes, calculating vote counting based on the direction of effect for adverse events and cosmetic outcomes.

### 2.6. Reporting Bias Assessment

No formal statistical methods were used to assess reporting bias, as no meta-analysis was performed. However, efforts were made to minimize potential bias by conducting a comprehensive search of multiple databases and clinical trial registries, supplemented by manual searching of reference lists of eligible studies. No additional unpublished studies were identified in clinical trial registries or other sources. Therefore, the risk of reporting bias is considered possible but not quantifiable, and conclusions should be interpreted with this limitation in mind.

### 2.7. Assessment of Certainty

The certainty of the evidence in the included studies was assessed narratively. Overall, the body of evidence was assessed as having low to moderate certainty. This assessment reflects the predominance of observational studies, the diversity of study populations and surgical techniques, and the observed differences in outcome assessment tools.

## 3. Results

### 3.1. Study Selection and Geographical Distribution

The systematic search identified a total of 1870 participants in ten studies (Supplementary Table S3) [11,22,28,29,30,31,32,33,34,35]. These studies covered a variety of breast surgery procedures, including breast reduction, mastopexy, quadrantectomy, mastectomy, and breast reconstruction. It should be pointed out that the included studies in this systematic review encompass procedures performed for both cosmetic and oncoplastic purposes. The outcomes therefore reflect the fact that the results were obtained across a broad spectrum of surgical objectives; as such, this variety of clinical contexts and patient populations should be taken into consideration when interpreting efficacy or complication rates.

Among the included studies, five were from the United States [28,30,31,33,35] involving a total of 1427 participants; this represented the vast majority of patients included in the review. In Europe, two studies were included, one from Italy [11] (N = 133) and another from the Netherlands [22] (N = 50). These studies provided valuable contextual information, allowing for a comparison of approaches to breast surgery between Europe and North America. In addition, three studies from Asia and the Middle East were included: one from Japan [32] (N = 100), one from Pakistan [29] (N = 100) and one from Saudi Arabia [34] (N = 60). Together, these studies covered all major continents, although the concentration of participants was clearly the highest in the United States.

The studied publications comprised randomized clinical trials (RCTs) and prospective and retrospective observational studies. Seven studies used a formal alternate traditional dressing, viz. two poliglecaprone 25 (Monocryl), or one n-butyl-2-cyanoacrylate (Histoacryl); in contrast, the other three cases did not use any formal comparison, i.e., exposure to 2-OCA was assessed in clinical conditions without control. This diverse methodology allows for the evaluation of Dermabond use in both controlled conditions and everyday clinical practice, increasing the extrapolation of results to a variety of breast surgical procedures.

The most common reason for exclusion was the fact that publications examined the Dermabond Prineo system [36,37,38,39,40,41]: while this contains Dermabond, it differed in terms of the design and function from pure 2-OCA. The inclusion of these studies could have introduced significant bias in the assessment of the efficacy and safety of pure Dermabond in breast surgery.

Publications reporting single cases or small series were also excluded [41,42,43,44,45]. While these papers may have valuable clinical observations, they did not meet the criteria for a primary study with a comparative control. Additionally, systematic reviews or letters to the Editor [39,46] were excluded as they did not provide primary data and were not consistent with the scope of our review.

Therefore, of the 22 assessed papers, 10 (45%) were included in the final analysis, covering 1870 patients. In total, 12 studies (55%) were excluded, mainly because they used the PRINEO system or were limited in nature (case report, letter, or review).

The included studies are summarized in Table 1*,* while a detailed description of the excluded studies is provided in Supplementary Table S3.

The overall geographic distribution indicates a clear concentration of studies in North America, with limited representation from other regions. Nevertheless, all the included studies meet the quality criteria and contribute significantly to the assessment of the efficacy and safety of 2-OCA in breast surgery, covering a variety of procedures such as breast reduction, mastopexy, breast reconstruction, quadrantectomy, mastectomy, and cosmetic and oncoplastic procedures.

The exact geographic distribution of studies meeting the inclusion criteria and the total sample size are presented in Supplementary Table S4.

### 3.2. Risk of Bias in Studies

The risk of bias was assessed with two complementary tools, depending on the study type. The Cochrane Risk of Bias 2 (RoB 2, version 2020) tool was applied for randomized studies, and Newcastle–Ottawa Scale (NOS) for retrospective and prospective and observational studies.

Each study was evaluated independently by two reviewers, who were unaware of each other’s results. If the evaluations differed, a third reviewer with greater methodological experience would make the decision. The third reviewer was also unaware of the previous evaluations. The final result was determined by consensus among the three reviewers.

To limit potential reviewer bias, the reviewers were unaware of the identity of the authors of the publications, the journals, or the country of origin of the studies. The analysis was performed manually using an original Excel spreadsheet provided by the Cochrane Collaboration for the RoB 2 tool, as well as a proprietary Excel template covering the full list of Newcastle–Ottawa Scale criteria.

The following classification rules have been adopted for the RoB 2 tool:

- If any one domain was rated as “high risk,” the entire study was classified as with a “high risk of bias”;
- If any one or more domains were rated as “some concerns,” the study was classified as “some concerns”;
- Studies in which all domains were rated as “low” were classified as with a “low risk of bias”.

For the Newcastle–Ottawa Scale, stars (★) were awarded according to the original assessment criteria in three domains: Selection, Comparability, and Outcome/Exposure.

Due to the specific nature of certain surgical procedures, not all items on the scale were completed if the information was not applicable (they were left blank). The final classification was determined according to the number of stars obtained:

- 0–5 stars—high risk of bias;
- 6–7 stars—moderate risk of bias;
- 8–9 stars—low risk of bias.

The evaluation of the results are presented in two forms: tabular (text and star rating) and graphical (in the form of a traffic light plot generated in a Cochrane Excel spreadsheet).

Additional data was not requested from the authors of the studies; the analysis was based solely on the information contained in the full text of the scientific articles. The GRADE system was not employed because the objective was to provide a qualitative assessment of the reliability of the evidence rather than a quantitative one.

A detailed description of the study and the scale used to assess the risk of systematic error are summarized in Table 2.

The observational studies included retrospective and prospective studies on wound healing after breast surgery. These studies vary with regard to their methodological quality and risk of bias: most demonstrated a moderate risk of bias, which is mainly due to limited control of confounders, incomplete follow-up documentation or missing data.

Two studies by Scott et al. [30,31] and Nakagawa et al. [32] exhibited moderate methodological quality, with relatively representative samples and verification of exposure/intervention; nevertheless, they demonstrated limited control of confounding factors, and the observation period was partially incomplete. In contrast, Nigro et al.’s [33] study was of slightly higher quality due to the greater detail in the follow-up documentation and additional control of confounding factors; nevertheless, it still exhibited a moderate risk of bias. The retrospective study by Alotaibi et al. (2022) [34] demonstrated a moderately high risk of bias mainly due to the lack of full exposure verification and the limited control of confounders.

The highest methodological quality among the reviewed papers was observed for the study by Francalancia et al. [35]; its low risk of bias allowed for a reliable assessment of complications. The paper provided complete perioperative documentation, the systematic control of confounding factors and complete postoperative follow-up.

A detailed assessment of methodological quality and risk of bias based on the Newcastle–Ottawa Scale (NOS) is presented in Supplementary Table S5.

Four studies were subjected to risk of bias analysis (RoB 2), covering five domains: the randomization process (D1), deviation from assigned intervention (D2), incompleteness of outcome data (D3), outcome measurement (D4), and selective reporting of outcomes (D5).

All four studies demonstrate a low risk of systematic error in D1 and D3. The most frequently identified limitations concerned D4 and D5, which were assessed as areas of concern in some cases. A moderate risk of error was associated with D2, but this did not significantly affect the overall assessment of the study quality.

Overall, three of the four studies were classified as having a low risk of systematic error, and the identified areas of uncertainty did not have any significant impact on the final conclusions.

However, it must be emphasized that the inherent difficulties in blinding patients and outcome assessors in surgical wound closure trials, combined with small sample sizes and short follow-up periods in several RCTs, limit the overall robustness of the evidence. The detailed results of the systematic bias risk assessment are presented in Supplementary Figure S1, generated using the Cochrane Excel Risk of Bias (RoB 2) tool.

### 3.3. Results of Overall Data

The systematic review summarized available clinical studies evaluating the efficacy and safety of 2-OCA-based tissue adhesives (Dermabond) compared to traditional wound closure methods; the review included randomized and observational studies covering breast reduction, mastopexy, reconstruction and selected cases of oncological treatment.

The descriptive analysis included data from all ten eligible studies, encompassing a total of 1870 patients. Despite the heterogeneity of the population, surgical techniques and observation time between studies, it was possible to estimate the incidence of selected complications in the intervention (2-OCA) and control (sutures) groups. Due to the heterogeneous nature of the studies, the analysis was narrative in nature.

Incidence of wound infections varied across studies, ranging from 0% to 5% in the 2-OCA groups and from 0% to 16% in the control groups. These differences were not significant; however, most analyses suggested a slightly lower risk of infection when using adhesive.

The wound dehiscence rates reported in comparative studies ranged from 0% to 7.6% for 2-OCA and from 0% to 14% for control groups. This potentially increased risk of wound edge separation in high-tension areas highlights that superficial adhesives cannot compensate for inadequate deep dermal support. The greatest difference was noted by Francalancia et al. [35] who reported a higher percentage of dehiscence in the adhesive group; this may reflect differences in closure technique or tissue tension distribution. More precisely, the insufficiency of the mechanical resistance of the adhesive often means that there is insufficient support for the deep layer of skin, which highlights that 2-OCA acts as a sealant for the epidermis and cannot compensate for the tension that should be managed by the more deeply located sutures.

Reported hematoma rates ranged from 1.5% to 9.7% in the 2-OCA groups and from 2.7% to 10.6% in the control groups. For seromas, the available data were limited to single retrospective studies, indicating an incidence of approximately 2.7% in the 2-OCA group, and no cases in the control groups.

However, tissue adhesives suggested a more favorable safety profile regarding the complications characteristic of breast surgery, such as nipple necrosis (3.67% for 2-OCA vs. 6.65%) or nipple epidermolysis (1.68% for 2-OCA vs. 2.65%). This may be due to better tissue perfusion and the absence of pressure characteristic of sutures. However, this relationship needs to be confirmed in prospective studies with greater statistical power.

Overall, while both groups achieved high scores for patient satisfaction and scar esthetics, some studies suggest a trend in favor of 2-OCA for both outcomes. However, it should be emphasized that the assessment used different assessment scales (i.e., Likert, VAS, and expert panels), which limits the comparison between studies. It is noteworthy that some studies (e.g., Scott, 2007 [30]) were unilateral (intra-patient design), which further increases the reliability of the comparison of effects. The observation period in the analyzed publications ranged from five days to twelve months. Gennari et al. (2004) found 2-OCA to enable shorter wound closure times and lower treatment costs, findings which have been confirmed by more recent clinical reports [11]. Hence, 2-OCA can be considered a valuable supplement to surgical techniques in breast surgery. This descriptive analysis is summarized in Table 3.

The characteristics of the individual studies, the type of treatment used, group sizes and the incidence of complications are summarized in Table 4. This table presents the key methodological parameters and clinical results for each study, allowing for a direct comparison of the efficacy and safety of 2-OCA tissue adhesive (Dermabond) compared to traditional wound closure methods.

### 3.4. Adverse Events and Allergic Reactions

Due to the inclusion and exclusion criteria, not all publications describing contact dermatitis and allergic reactions after the use of Dermabond tissue adhesive were included in the review. Our analysis of available case reports and observational studies indicates that such adverse reactions are rare, but may take the form of both mild contact dermatitis and more severe allergic reactions. A collection of studies describing such events is presented in Table 5.

## 4. Discussion

The nature of the corpus of articles included in this review unfortunately was insufficient for a full meta-analysis. The number of eligible studies was limited (n = 10), thus resulting in a lack of statistical power and increasing the risk of erroneous estimates, and the studies themselves demonstrated considerable methodological heterogeneity: they covered different procedures (oncological, esthetic, and reconstructive) and different techniques, and lacked a clearly described distribution across populations. The clinical status of the patients also varied; this is an important consideration as oncology patients demonstrate different wound healing processes to healthy individuals. Considerable variation was also observed in relation to the length of postoperative follow-up, ranging from 5 days to 12 months, which made it impossible to compare results within a uniform time interval. Furthermore, the reporting of results was inconsistent: some authors provided percentages, while others provided only numbers without reference to the denominator, which made reliable aggregation difficult. Most studies did not present standard effect measures such as RR, OR, or RD, which form the basis of quantitative meta-analysis. The studies used different methods of esthetic assessment—the authors used different types of scales, frequently with unverified accuracy and reliability. The assessments came from patients, surgeons, and external observers. The inclusion and exclusion criteria varied between studies, and the sample size ranged from approximately 20 to over 500 patients. This range created a strong weighting effect and could distort the proportions of data in the classic meta-analysis model.

It should also be emphasized that many studies lacked consistent and complete raw data; as such, no accurate statistical analysis could be performed. Therefore, the review was based on a reliable descriptive analysis.

Despite the lack of a quantitative meta-analysis, the systematic review provides a comprehensive overview of the use of Dermabond 2-cyanoacrylate in breast surgery. While the findings are diverse, they provide an insight into recurring clinical trends and the safety of wound closure methods. It was found that Dermabond showed a comparable safety profile to suturing techniques, and in some cases, demonstrated more favorable healing parameters and less tissue tension.

The descriptive analysis also included an assessment of potential esthetic benefits. Many studies reported good or very good healing quality and high patient satisfaction, which is particularly important in plastic and reconstructive breast surgery. In addition, the paper presents a summary of possible complications associated with Dermabond use, which may be a valuable reference point for surgeons operating in different clinical settings.

This is the first systematic review to exclusively examine the use of Dermabond in breast surgery, as similar reviews of tissue adhesives have focused on Dermabond PRINEO systems; this is a different system that contains an additional polymer mesh [40].

The synthesis provides important information on current clinical practices and preferences in wound closure in breast surgery. The analysis identified areas requiring methodological improvement, such as the standardization of criteria for reporting complications, the length of follow-up, the methods of esthetic assessment, and indications for the use of tissue adhesive. These results have real implications for the design of future randomized clinical trials, which should consider the specific nature of breast wounds, patients’ expectations, and the impact of oncological burden on the healing process.

This review represents an initial step toward developing broader guidelines for the use of Dermabond in breast surgery and may support surgeons in their clinical decision making. The collected data can serve as a consistent reference point, facilitating the interpretation of results and the planning of further research.

It is also worth emphasizing the significance of tissue adhesives in breast cancer surgery, especially in the context of postoperative outcomes. In a retrospective study, De Luca et al. compared the use of 2-OCA with fibrin sealant and the standard technique without adhesive after axillary lymphadenectomy [47].

Although cyanoacrylate did not significantly affect the overall incidence of seroma, a clear reduction in the duration of axillary drainage and a reduction in the percentage of postoperative infections were demonstrated. These results indicate that the use of cyanoacrylate adhesive may be a valuable adjunctive strategy to reduce complications that may delay the initiation of adjuvant therapy [47].

Despite these promising findings, several limitations of this review must be acknowledged. First, the literature search was limited to articles published in English, which may have led to the omission of relevant studies in other languages. Second, unpublished literature and the “gray literature,” including conference reports, research protocols, and master’s theses, were excluded. Third, only selected databases (PubMed, Embase, Web of Science, ClinicalTrials.gov, and WHO ICTRP) were searched. Fourth, due to the considerable heterogeneity of the studies and the reported results, it was not possible to perform a formal quantitative meta-analysis, which limited the possibilities for a synthetic assessment of the effects of the intervention. Finally, despite efforts to comprehensively search the literature and manually check references, the risk of omitting unpublished studies or the presence of bias in the reporting of results in the original studies cannot be ruled out.

On the basis of a systematic review of the published literature, 2-OCA (Dermabond) can be considered a worthwhile alternative to traditional sutures for wound closure in breast surgery. Dermabond shows comparable efficacy and safety to traditional sutures, while also contributing to reduced patient discomfort and simplified postoperative care [31].

Other randomized studies in various branches of surgery have also obtained similar results, which confirm the lack of significant differences in the long-term cosmetic assessments and patient satisfaction between Dermabond and controls [30]. It is worth emphasizing that the surgeon’s decision regarding the choice of wound closure method should be made after consultation with the patient, taking into account their individual expectations and preferences, in order to achieve the optimal cosmetic and functional effect. Surgeons should be able to use both Dermabond and classic dressings in breast surgery as appropriate, depending on the clinical context and the individual preferences of the patient [48].

## 5. Conclusions

Narrative synthesis suggests that Dermabond may offer a comparable safety profile to traditional sutures regarding infection and hematoma rates. However, surgeons should be mindful of a potentially higher risk of wound dehiscence in high-tension areas. Patient satisfaction is generally higher, and cosmetic results are comparable or slightly better than with sutures, especially in long-term follow up. Although allergic reactions and contact dermatitis are rare, they should be considered when choosing the optimal wound closure method.

Despite these positive results, further well-designed studies, including larger randomized controlled trials with extended follow-ups, are needed to more accurately assess rare complications, long-term cosmetic outcomes and patient-reported satisfaction, and to formulate future recommendations. Although several studies consistently report favorable cosmetic outcomes and reduced operative time with octyl-2-cyanoacrylate (2-OCA), the lack of randomized controlled trials with standard endpoints limits the strength of the conclusions. Therefore, the results should be interpreted with caution, as the available studies have numerous methodological limitations. New, well-designed, high-quality trials are needed to enable a reliable meta-analysis and strengthen the evidence base in this area.

In upcoming randomized controlled trials (RCT), it was suggested that procedures should ideally be separated by type (e.g., reduction, augmentation, or reconstruction) to reflect the wide variation in tissue tension and biomechanical requirements.

As adhesives and sutures are associated with similar complication rates and comparable esthetic results, the choice of wound closure technique depends on the individual patient and her personal preferences, as well as the clinical context.

## Figures and Tables

**Table 1:** Studies included in the systematic review.

No	Author (Year)	Country	Study Type	N	Surgical Context	Comparator
**1**	Gennari et al. (2004) [11]	Italy	RCT	133	Quadrantectomy, mastectomy, benign excisions	2-OCA vs. subcutaneous sutures
**2**	Scott et al. (2005) [31]	USA	Observational (retrospective)	518	Breast reduction	2-OCA vs. Monocryl (poliglecaprone 25)
**3**	Scott et al. (2007) [30]	USA	Observational (retrospective)	255	Bilateral breast reduction	2-OCA vs. regular sutures
**4**	Nipshagen et al. (2008) [22]	Netherlands	RCT	50	Breast reduction	2-OCA vs. Monocryl (poliglecaprone 25)
**5**	Koonce et al. (2015) [28]	USA	RCT	20	Breast reduction, mastopexy, abdominoplasty	2-OCA vs. Histoacryl (n-butyl-2-cyanoacrylate) sutures
**6**	Majeed et al. (2018) [29]	Pakistan	RCT	100	Breast surgery (various procedures)	2-OCA vs. sutures
**7**	Nakagawa et al. (2018) [32]	Japan	Observational	100	Breast reconstruction	No formal comparator
**8**	Nigro et al. (2020) [33]	USA	Observational (prospective)	102	Breast reconstruction	No formal comparator
**9**	Alotaibi et al. (2022) [34]	Saudi Arabia	Observational (retrospective)	60	Breast reduction and mastopexy	No formal comparator
**10**	Francalancia et al. (2025) [35]	USA	Observational (retrospective)	532	reduction mammoplasty	2-OCA vs. Traditional dressing

RCT = randomized controlled trial, 2-OCA = 2-octyl cyanoacrylate (main ingredient in Dermabond tissue adhesive), Dermabond = commercial name of tissue adhesive containing pure 2-octyl cyanoacrylate, USA = United States of America, and UK = United Kingdom.

**Table 2:** Tools used to assess bias risk depending on the type of study.

No	Author (Year)	Study Type	Risk of Bias Tool	Domains/Components Assessed	Overall Risk of Bias
**1**	Gennari et al. (2004) [11]	RCT	RoB 2	Randomization, deviations, missing data	Low
**2**	Scott et al. (2005) [31]	Observational (retrospective)	NOS	Selection, comparability, outcome assessment	Moderate
**3**	Scott et al. (2007) [30]	Observational (retrospective)	NOS	Selection, comparability, outcome assessment	Moderate
**4**	Nipshagen et al. (2008) [22]	RCT	RoB 2	Randomization, deviations, missing data	Moderate
**5**	Koonce et al. (2015) [28]	RCT	RoB 2	As above	Low
**6**	Majeed et al. (2018) [29]	RCT	RoB 2	As above	Low
**7**	Nakagawa et al. (2018) [32]	Observational	NOS	Selection, outcome assessment	Moderate
**8**	Nigro et al. (2020) [33]	Observational (prospective)	NOS	Selection, comparability, outcome assessment	Moderate
**9**	Alotaibi et al. (2022) [34]	Observational (retrospective)	NOS	Selection, comparability, outcome assessment	High
**10**	Francalancia et al. (2025) [35]	Observational (retrospective)	NOS	Selection, comparability, outcome assessment	Low

RCT = randomized controlled trial, RoB 2—risk of bias 2 tool, and NOS = Newcastle–Ottawa Scale.

**Table 3:** Summarized data from the ten studies meeting the inclusion criteria.

Outcome	Events (Interv)	Total (Interv)	Mean Rate (Interv)	Events (Ctrl)	Total (Ctrl)	Mean Rate (Ctrl)	No. of Studies	Notes/Interpretation
**Infection**	19	816	**2.33%**	17	560	**3.04%**	5	Similar infection rates between groups; slight advantage for Dermabond.
**Dehiscence**	50	697	**7.17%**	14	476	**2.94%**	5	Higher percentage of separations with Dermabond in some studies (especially Francalancia, 2025) [35].
**Hematoma**	29	627	**4.63%**	19	376	**5.05%**	3	Comparable results—no clear advantage of any method.
**Seroma**	7	255	**2.75%**	0	255	**0.00%**	2	Limited data; possible underestimation.
**Nipple necrosis**	23	627	**3.67%**	25	376	**6.65%**	3	Trend towards less necrosis with Dermabond.
**Nipple epidermolysis**	6	358	**1.68%**	3	113	**2.65%**	2	A slight difference; requires further research.
**Satisfaction (Likert/score)**	—	—	**~4.66 ± 0.90**	—	—	**~7.22 ± 0.70**	7–10	Various scales; generally high patient satisfaction—better for Dermabond.
**Cosmetic score**	—	—	**8.35 ± 0.05**	—	—	**8.05 ± 0.05**	7–10	High esthetic ratings in both groups. No significant difference

Interv—intervention (2-OCA); Ctrl—control (sutures);

**Table 4:** Characteristics and key outcomes of studies comparing 2-octyl cyanoacrylate (Dermabond) with conventional wound closure techniques in breast surgery.

Study (Year)	Country	Design	N (Interv/Ctrl)	Surgery Type	Main Outcomes (2-OCA vs. Control)	Follow-Up	Key Findings/Comments
**Gennari et al. (2004) [11]**	Italy	RCT	69/64	Quadrantectomy, mastectomy	Infection NR; Dehiscence NR; Satisfaction 9.5 vs. 7.45 ^O^ Cosmetic 8.8 vs. 8.8 ^X^	5 d–12 mo	No major complications; similar cosmetic results, higher satisfaction and faster closure with 2-OCA.
**Scott et al. (2005) [31]**	USA	Retrospective	103/113	Reduction mammaplasty	Inf 0% vs. 0.9% ^X^ Dehiscence 3.9% vs. 4.4% ^X^ Hematoma 9.7% vs. 10.6% ^X^ Nipple necrosis 19% vs. 22% ^X^	6–12 mo	Comparable complication rates; no increase in wound healing issues; earlier series used sutures.
**Scott et al. (2007) [30]**	USA	Retrospective (single-arm)	255/–	Reduction mammaplasty	Inf 1.2% ^X^ Dehiscence 2.0% ^X^ Hematoma 5.9% ^X^ Nipple necrosis 0.8% ^X^	6–12 mo	High satisfaction and low complication rate with 2-OCA; operative time reduced (120 vs. 102 min).
**Nipshagen et al. (2008) [22]**	Netherlands	Bilateral intra-patient RCT	50/50	Reduction mammaplasty	Infection 0% vs. 0% ^X^; Dehiscence 0% vs. 0% ^X^ Satisfaction 7.2 vs. 7.0 ^X^ Cosmetic 7.9 vs. 7.3 ^O^	6 mo	Adhesive preferred by patients and panelists; less itching and better scar appearance.
**Koonce et al. (2015) [28]**	USA	RCT (intra-patient)	20/20	Reduction mammaplasty, mastopexy	Infection 5% vs. 5% ^X^; Dehiscence 5% vs. 0% ^X^ Satisfaction similar ^X^ Scar width comparable ^X^	12 mo	Closure time reduced; one allergic and four mild adverse reactions observed.
**Majeed et al. (2018) [29]**	Pakistan	RCT	50/50	Lumpectomy	Infection 4% vs. 16% ^O^ Dehiscence 2% vs. 14% ^O^	7 d	Significantly fewer complications and faster closure in 2-OCA group (*p* < 0.05).
**Nakagawa et al. (2018) [32]**	Japan	Retrospective	100/–	Reconstruction, donor site closure	CD 7% ^X^	Variable	Allergic reactions mainly after repeated exposure; all mild and self-limited.
**Nigro et al. (2020) [33]**	USA	Prospective	102/–	Cosmetic and reconstructive breast surgery	Allergic/CD 14% ^X^	≤1 week	All allergic to Dermabond also reacted to LiquiBand; symptoms resolved with topical therapy.
**Alotaibi et al. (2022) [34]**	Saudi Arabia	Retrospective	60/–	Mastopexy, reduction	CD 6.6% ^X^	2 w–6 mo	Type IV hypersensitivity; post-inflammatory hyperpigmentation up to 6 mo.
**Francalancia et al. (2025) [35]**	USA	Retrospective cohort	269/263	Reduction mammaplasty	Infection 4.8% vs. 2.7% ^X^ Dehiscence 7.6% vs. 0.8% ^O^ Hematoma 1.5% vs. 2.7% ^X^ Nipple necrosis 0.4% vs. 0% ^X^	Mean 183 d	Higher dehiscence in 2-OCA group; adjusted *p* < 0.001. No other differences observed.

2-OCA—2-octyl cyanoacrylate; RCT—randomized controlled trial; NA—not available/not applicable; NR—not reported; Interv—intervention group (Dermabond); Ctrl—control group (traditional suture closure); CI—confidence interval; SD—standard deviation; mo—months; w—weeks; d—days; *p*—*p*-value; AE—adverse event; and CD—contact dermatitis. ^O^—significant statistical difference; ^X^—no statistically significant difference.

**Table 5:** Reported cases of allergic reactions/contact dermatitis (AEs).

No	Author (Year)	Country	Publication Type	n/N	Surgical Context	Description of AE
**1**	Perry and Sosin (2009) [43]	USA	Case report	1/1	Implant replacement + bilateral mastopexy	Severe allergic reaction to Dermabond
**2**	Howard and Downey (2010) [45]	USA	Case report	2/2	Bilateral breast reduction	Contact dermatitis to Dermabond
**3**	Ricci et al. (2014) [44]	USA	Case report	1/1	Bilateral skin-sparing mastectomy + reconstruction	Diffuse cutaneous allergic reaction to Dermabond
**4**	Nakagawa et al. (2018) [32]	Japan	Observational (retrospective)	7/100	Breast reconstruction	Contact dermatitis caused by Dermabond Advanced
**5**	Nigro et al. (2020) [33]	USA	RCT	14/102	Reconstructive breast surgery	Incidence of dermatitis after 2-OCA exposure
**6**	Alotaibi et al. (2022) [34]	Saudi Arabia	Observational (retrospective)	4/60	Reduction mammoplasty, mastopexy	Type IV hypersensitivity reaction to Dermabond

n/N—cases of allergy or contact dermatitis in relation to the entire study group.

## Data Availability

The original contributions presented in this study are included in the article and Supplementary Materials. Further inquiries can be directed to the corresponding author.

## Supplementary Materials

The following supporting information can be downloaded at https://www.mdpi.com/article/10.3390/jcm15062462/s1, Figure S1: Detailed results of systematic bias for randomized controlled trials using the RoB2 tool [11,22,28,29]. Table S1: PRISMA Checklist. Table S2: Search strategy and eligibility criteria used in the systematic review. Table S3: Studies excluded from systematic review [36,37,38,39,40,41,42,43,44,45,46,49]. Table S4: Geographic distribution of included studies according to country of origin and total sample size. Table S5: Assessment of methodological quality and risk of systematic error in observational studies on 2-OCA tissue adhesive breast surgery—Newcastle–Ottawa Scale (NOS) [30,31,32,33,34,35].

## Abbreviations

The following abbreviations are used in this manuscript:

2-OCA	Octyl 2-cyanoacrylate (Dermabond)
RCT	Randomized controlled trials
RoB 2	Cochrane Risk of Bias 2 tool
NOS	Newcastle–Ottawa Scale
PRINEO	Wound closure system combining polymer mesh with 2-OCA
TE	Tissue expander
NSM	Nipple-sparing mastectomy
SSM	Skin-sparing mastectomy
AE(s)	Allergic reaction(s)/contact dermatitis
