# Supplementary material for: Safety and Efficacy of Octyl 2-Cyanoacrylate (Dermabond) in Breast Surgery: A Systematic Review
Source: J Clin Med. 2026 Mar 23;15(6):2462. doi: 10.3390/jcm15062462 (PMC13026982; doi:10.3390/jcm15062462)
Supplement: Supplementary file 1 [file jcm-15-02462-s001.zip › jcm-4189338-supplementary.pdf]

Safety and Efficacy of octyl 2-cyanoacrylate (Dermabond) in Breast Surgery: a Systematic Review

Supplementary Materials

Supplementary Table S1. PRISMA Checklist

Section and Topic	Item #	Checklist item	Location where item is reported
**TITLE**			
Title	1	Identify the report as a systematic review.	Title
**ABSTRACT**			
Abstract	2	See the PRISMA 2020 for Abstracts checklist.	Abstract
**INTRODUCTION**			
Rationale	3	Describe the rationale for the review in the context of existing knowledge.	paragraphs 1–7 of the introduction
Objectives	4	Provide an explicit statement of the objective(s) or question(s) the review addresses.	End of introduction, final paragraph
**METHODS**			
Eligibility criteria	5	Specify the inclusion and exclusion criteria for the review and how studies were grouped for the syntheses.	Eligibility criteria detailed in the narrative methods section and Supplementary Table 1
Information sources	6	Specify all databases, registers, websites, organisations, reference lists and other sources searched or consulted to identify studies. Specify the date when each source was last searched or consulted.	2.1. Sources of Information and Search Strategy and Supplementary Table 1
Search strategy	7	Present the full search strategies for all databases, registers and websites, including any filters and limits used.	2.1. Sources of Information and Search Strategy; detailed descriptions of queries for PubMed/Embase/WoS/CT.gov/ICTRP + Supplementary Table 1.
Selection process	8	Specify the methods used to decide whether a study met the inclusion criteria of the review, including how many reviewers screened each record and each report retrieved, whether they worked independently, and if applicable,	‘Section 2.2. Research Selection, Data Gathering and Collection

Section and Topic	Item #	Checklist item	Location where item is reported
		details of automation tools used in the process.	Process
Data collection process	9	Specify the methods used to collect data from reports, including how many reviewers collected data from each report, whether they worked independently, any processes for obtaining or confirming data from study investigators, and if applicable, details of automation tools used in the process.	Paragraph “Research selection, data gathering, and collection process”
Data items	10a	List and define all outcomes for which data were sought. Specify whether all results that were compatible with each outcome domain in each study were sought (e.g. for all measures, time points, analyses), and if not, the methods used to decide which results to collect.	‘Data items’
	10b	List and define all other variables for which data were sought (e.g. participant and intervention characteristics, funding sources). Describe any assumptions made about any missing or unclear information.	No additional variables beyond outcomes were pre-specified
Study risk of bias assessment	11	Specify the methods used to assess risk of bias in the included studies, including details of the tool(s) used, how many reviewers assessed each study and whether they worked independently, and if applicable, details of automation tools used in the process.	Section ‘Risk of systematic bias in particular studies’ and Supplementary Table 4 and Supplementary Figure 1 ,
Effect measures	12	Specify for each outcome the effect measure(s) (e.g. risk ratio, mean difference) used in the synthesis or presentation of results.	Section ‘Effect measures and synthesis methods’-Only narrative effect descriptions were provided
Synthesis methods	13a	Describe the processes used to decide which studies were eligible for each synthesis (e.g. tabulating the study intervention characteristics and comparing against the planned groups for each synthesis (item #5)).	Section 2.5. Effect Measures and Synthesis Methods and Section 4. Discussion (paragraph 1-2)
	13b	Describe any methods required to prepare the data for presentation or synthesis, such as handling of missing summary statistics, or data conversions.	No statistical data transformations or imputations were performed
	13c	Describe any methods used to tabulate or visually display results of individual studies and syntheses.	Tables 2-4 and ‘Results’ section.
	13d	Describe any methods used to synthesize results and provide a rationale for the choice(s). If meta-analysis was performed, describe the model(s), method(s) to identify the presence and extent of statistical heterogeneity, and software package(s) used.	Only narrative/partial quantitative summaries; no meta-analysis
	13e	Describe any methods used to explore possible causes of heterogeneity among study results (e.g. subgroup analysis, meta-regression).	No formal analyses. description in “Justification for inability to perform meta-analysis” (variety of methods, populations, observation time).
	13f	Describe any sensitivity analyses conducted to assess robustness of the synthesized results.	None
Reporting bias assessment	14	Describe any methods used to assess risk of bias due to missing results in a synthesis (arising from reporting biases).	Section “Reporting bias assessment.”
Certainty assessment	15	Describe any methods used to assess certainty (or confidence) in the body of evidence for an outcome.	Section “Assessment of certainty.”
**RESULTS**			

Section and Topic	Item #	Checklist item	Location where item is reported
Study selection	16a	Describe the results of the search and selection process, from the number of records identified in the search to the number of studies included in the review, ideally using a flow diagram.	Section “Study selection” and PRISMA flow diagram (Figure 1).
	16b	Cite studies that might appear to meet the inclusion criteria, but which were excluded, and explain why they were excluded.	Supplementary Table 2 with justification for exclusion.
Study characteristics	17	Cite each included study and present its characteristics.	Table 2, section ‘Study selection and geographical distribution’, Supplementary Table 3
Risk of bias in studies	18	Present assessments of risk of bias for each included study.	‘Risk of bias in studies’. Detailed NOS ratings are available in Supplementary Table 4, and RoB-2 domain-level results in Supplementary Figure 1
Results of individual studies	19	For all outcomes, present, for each study: (a) summary statistics for each group (where appropriate) and (b) an effect estimate and its precision (e.g. confidence/credible interval), ideally using structured tables or plots.	Tables 3-4 and ‘Result’
Results of syntheses	20a	For each synthesis, briefly summarise the characteristics and risk of bias among contributing studies.	Results section and Tables 2, Supplementary Table 4 and Supplementary Figure 1.
	20b	Present results of all statistical syntheses conducted. If meta-analysis was done, present for each the summary estimate and its precision (e.g. confidence/credible interval) and measures of statistical heterogeneity. If comparing groups, describe the direction of the effect.	‘Results of No meta-analytic summary measures were produced.
	20c	Present results of all investigations of possible causes of heterogeneity among study results.	No formal analyses; description of heterogeneity in the Discussion section.
	20d	Present results of all sensitivity analyses conducted to assess the robustness of the synthesized results.	None
Reporting biases	21	Present assessments of risk of bias due to missing results (arising from reporting biases) for each synthesis assessed.	Section ‘Reporting bias assessment.’
Certainty of evidence	22	Present assessments of certainty (or confidence) in the body of evidence for each outcome assessed.	Section ‘Assessment of certainty’, no GRADE
**DISCUSSION**			
Discussion	23a	Provide a general interpretation of the results in the context of other evidence.	Section 4. Discussion (paragraphs 4-5)
	23b	Discuss any limitations of the evidence included in the review.	paragraphs and the discussion on heterogeneity.
	23c	Discuss any limitations of the review processes used.	Section 4. Discussion (paragraph 9, limitations)

Section and Topic	Item #	Checklist item	Location where item is reported
	23d	Discuss implications of the results for practice, policy, and future research.	Section 4. Discussion and Section 5. Conclusions
**OTHER INFORMATION**			
Registration and protocol	24a	Provide registration information for the review, including register name and registration number, or state that the review was not registered.	Abstract and Material and Methods
	24b	Indicate where the review protocol can be accessed, or state that a protocol was not prepared.	Abstract and Material and Methods
	24c	Describe and explain any amendments to information provided at registration or in the protocol.	not applicable
Support	25	Describe sources of financial or non-financial support for the review, and the role of the funders or sponsors in the review.	Section “Funding”
Competing interests	26	Declare any competing interests of review authors.	Section "Conflict of interest"
Availability of data, code and other materials	27	Report which of the following are publicly available and where they can be found: template data collection forms; data extracted from included studies; data used for all analyses; analytic code; any other materials used in the review.	No data sets or additional materials have been deposited or made publicly available. Only Supplementary Tables 1-5 and Supplementary Figure 1

Supplementary Table S2. Search strategy and eligibility criteria used in the systematic review.

Items	Specification
Date of search	10, 17, 20.03.2023 (updated 28.03.2024 and 3.09.2025)
Databases and other sources searched	PubMed, Web of Science, ClinicalTrials.Org and hand searches from included studies data
Search terms used	„octyl cyanoacrylate”, „Dermabond”, „2-octyl cyanoacrylate”, „mammaplasty” „breast reconstruction”, „breast implantation”, „breast prosthesis”, „breast implant”, „mastopexy”, „breast conservation surgery”, „breast oncoplasty”,„oncoplastic surgery”, „breast deformity”

Inclusion and exclusion criteria	Inclusion criteria included observational or experimental original articles or systematic reviews of these studies published in peer-reviewed journals indexed to the aforementioned databases. Non-English manuscripts and abstracts were excluded from the review.
Selection process	Selection process was conducted by authors:
Time frame covered by the search	January 2004 - September 2025

Supplementary Table S3. Studies excluded from systematic review.

No	Author (year)	Country	Study type	N	Surgical context	Justification for exclusion
1	Perry and Sosin (2009) [43]	USA	Case report	1	Implant replacement with mastopexy	Single case of allergic reaction
2	Howard and Downey (2010) [45]	USA	Case report	2	Breast reduction	Single cases of allergic reaction
3	Colwell et al. (2010) [42]	USA	Case report	8	Nipple-sparing mastectomy	Technical article, no comparative data available.
4	Richter et al. (2012) [36]	Sweden/Belgium	RCT	83	PRINEO vs sutures	The PRINEO system was used
5	Blondeel et al. (2014) [37]	Belgium	RCT	79	PRINEO vs sutures	The PRINEO system was used

6	Ricci et al. (2014) [44]	USA	Case report	1	Mastectomy with reconstruction	Single case of a allergic reaction.
7	Knackstedt et al. (2015) [41]	USA	Case series	1	Breast reduction	Single case of allergic reactions
8	Lee et al. (2018) [38]	USA	RCT	21	PRINEO vs. intradermal sutures	The PRINEO system was used
9	Libretti et al. (2023) [39]	Italy	Systematic review	-	Various fields of surgery (orthopedics, gynecology, plastic surgery, including several cases of mammoplasty) with PRINEO	The PRINEO system was used
10	Kulkarni et al. (2025) [40]	UK	Systematic review	-	Review of studies with PRINEO and Dermabond	The PRINEO system was used
11	Mangano et al. (2008) [46]	Italy	Letter to the Editor/Commentary	-	No clinical data available; comment on Nipshagen et al. (2008)	Not an original study or a systematic review.
12	Aburakawa et al. (2025) [49]	Japan	Quasi-RCT	18	Two-stage breast reconstruction (TE, NSM/SSM) PRINEO vs Surgical sutures	The PRINEO system was used

RCT = Radomized Controlled Trial,

2-OCA = 2-Octyl Cyanoacrylate (main ingredient in Dermabond tissue adhesive),

Dermabond = Commercial name of tissue adhesive containing pure 2-octyl cyanoacrylate,

PRINEO = Wound closure system combining polymer mesh with 2-OCA (a product other than Dermabond),

USA = United States of America

UK= United Kingdom

Selection domain: representativeness of the sample, recruitment method, confirmation of exposure/intervention, lack of pre-intervention results.

Comparability domain: control of confounding factors (age, type of procedure, surgical technique).

Outcome/Exposure domain: method of outcome assessment, length and completeness of follow-up

Supplementary Table S4. Geographic distribution of included studies according to country of origin and total sample size.

Region	Number of Studies	Total Sample Size (n)
USA	5	1427
Italy	1	133
Japan	1	100
Pakistan	1	100
Saudi Arabia	1	60
Netherlands	1	50
**SUMMARY**	**10**	**1870**

Supplementary Table S5. Assessment of methodological quality and risk of systematic error in observational studies on 2-OCA tissue adhesive breast surgery – Newcastle–Ottawa Scale (NOS).

			Selection				Comparability		Outcome/Eposure				
**No**	**Author (year)**	**Study Type**	**Exposed**	**Non-exposed**	**Ascertainment of exposure**	**Start without outcome present**	**Major factor**	**Addition factor**	**Outcomes assessment**	**Follow-up length**	**Adequancy of outcome**	**Summary**	**Overall level of bias risk**
**2**	Scott et al. (2005) [31]	R	★	★	★	★	★		★			6	Moderate
**3**	Scott et al. (2007) [30]	R	★	★	★	★	★		★			6	Moderate
**7**	Nakagawa et al. (2018) [32]	P	★	★	★	★	★		★			6	Moderate
**8**	Nigro et al. (2020) [33]	P	★	★	★	★	★		★	★		7	Moderate
**9**	Alotaibi et al. (2022) [34]	R	★	★		★	★		★			5	High
**10**	Francalancia et al. (2025) [35]	R	★	★	★	★	★	★	★	★	★	9	Low

Supplementary Figure S1. Detailed results of systematic bias for randomized controlled trials using the RoB2 tool.

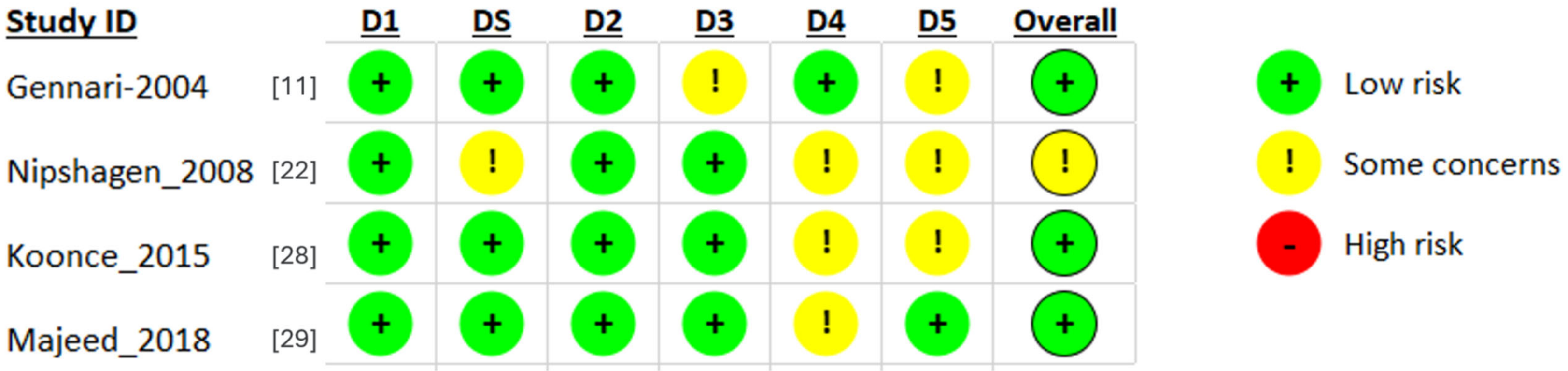

D1 Randomisation process
DS Bias arising from period and carryover effects
D2 Deviations from the intended interventions
D3 Missing outcome data
D4 Measurement of the outcome
D5 Selection of the reported result
